# Unexpected electronic structure of the alloyed and doped arsenene sheets: First-Principles calculations

**DOI:** 10.1038/srep29114

**Published:** 2016-07-04

**Authors:** Ming-Yang Liu, Yang Huang, Qing-Yuan Chen, Chao Cao, Yao He

**Affiliations:** 1 Department of Physics, Yunnan University, Kunming 650091, China; 2 Department of Physics, Hangzhou Normal University, Hangzhou 310036, China

## Abstract

We study the equilibrium geometry and electronic structure of alloyed and doped arsenene sheets based on the density functional theory calculations. AsN, AsP and SbAs alloys possess indirect band gap and BiAs is direct band gap. Although AsP, SbAs and BiAs alloyed arsenene sheets maintain the semiconducting character of pure arsenene, they have indirect-direct and semiconducting-metallic transitions by applying biaxial strain. We find that B- and N-doped arsenene render p-type semiconducting character, while C- and O-doped arsenene are metallic character. Especially, the C-doped arsenene is spin-polarization asymmetric and can be tuned into the bipolar spin-gapless semiconductor by the external electric field. Moreover, the doping concentration can effectively affect the magnetism of the C-doped system. Finally, we briefly study the chemical molecule adsorbed arsenene. Our results may be valuable for alloyed and doped arsenene sheets applications in mechanical sensors and spintronic devices in the future.

Two-dimensional (2D) materials have become emerging materials for electronics^1^,^2^,^3^,^4^. Due to graphene, group IV elemental monolayers silicene and germanene firstly attract tremendous attention^5^,^6^,^7^. The electronic properties of silicene and germanene are analogous to graphene, namely the Dirac-like gapless electronic properties, which also has greatly impeded their applications in electronics^7^. In the meanwhile, transition metal dichalcogenides, garnering a lot of attentions, their mobility is limited owing to the presence of heavy transition metal atoms even if they possess intrinsic band gaps^8^,^9^. In recent two years, group V elemental monolayers phosphorus have been found that they have two typical structures, puckered and buckled, referred as black phosphorene and blue phosphorene, both of them exhibit a high carrier mobility and significant band gap^10^,^11^,^12^,^13^,^14^. Whereas, applying to optoeletronic devices may be restricted, especially photoresponse in the blue UV range, since the fundamental band gap of phosphorene is smaller than 2.0 eV^8^.

With an intensive study for phosphorene, in the same group, the monolayer arsenic has been investigated, recently^8^,^9^,^15^,^16^,^17^,^18^,^19^. Similar to the phosphorene, the stability of two structures puckered and buckled arsenene has already been shown by the phonon spectra and cohesive energy, and the buckled arsenene is slightly more stable than puckered one^19^. According to the calculations by HSE06 hybrid functional, an indirect band gap with larger than 2.0 eV is obtained in buckled arsenene^8^,^17^. Theoretically, it has been reported that the strain can induce metal-semiconductor and indirect-direct band-gap transitions in arsenene using First-principles calculations^8^,^9^,^17^,^19^,^20^. Very recently, the electronic structures and magnetic properties of arsenene are also studied via impurity doping^21^. Furthermore, in experiment, the arsenene is very possible to be manufactured by exfoliating gray arsenic like black phosphorene due to the weak interaction between layers in bulk gray arsenic^19^. Such wide band gap and strain-modulated electronic structure are promising for applying to optoeletronic devices and mechanical sensors^8^. However, the more abundant properties of arsenene need be thoroughly investigated, for instance its alloys, different doping concentrations and molecule adsorption systems.

In this work, firstly, we investigate the stability and electronic structure of buckled arsenene and its alloys based on the density functional theory (DFT) calculations, since there are some unexpected results by alloying the group V elements themselves both in theories and experiments^17^,^22^. In alloyed systems, we use the buckled arsenene model, but substituting half the As atoms with N, P, Sb and Bi atoms, respectively. Our calculations reveal that the electronic structure of four alloys is sensitive to diverse strain yielding the semiconductor-metal and indirect-direct band gap transitions. Next, the electronic structure of arsenene doped with B, C, O, F, N, P, Sb and Bi eight atoms is also studied. Particularly, it has been found that B-and N-doped arsenene exhibit p-type doping character, but C and O dopants induce the metallic character to change. A bipolar spin-gapless semiconductor is gained by applying an appropriate external electric field for the C-doped arsenene because of the spin-polarization asymmetry. Then the effect of doping concentration is also discussed for the doped systems. Finally, we briefly study the chemical molecule adsorbed arsenene. We hope that our interesting findings will advance more experimental and theory investigations.

## Methods

We performed our First-principle calculations based on density functional theory (DFT) as implemented in the Vienna Ab Initio Simulation Package (VASP)^23^ codes. To avoid the inter-layer interaction between layers and simulate period boundary conditions, a vacuum of about 18 Ǻ along the z direction was employed. The unitcell was used in pristine and alloyed arsenene calculations, 2 × 2 × 1, 3 × 3 × 1, 4 × 4 × 1 and 8 × 8 × 1 supercells were used in doped systems, and the adsorbed arsenene used 4 × 4 × 1 supercell. The large doping ratio cases were mainly studied in this paper since we obtained a semiconductor-metal transition for the C-doped system. Unless specified otherwise, for the exchange correlation interaction, we utilized the Perdew-Burke-Ernzerhof (PBE)^24^ for most calculations. The cutoff energy of 500 eV was used for the plane wave basis. We adopted a 11 × 11 × 1 *k*-grid mesh in the Brillouin zone. For ionic relaxations, the convergence criterion between two consecutive steps in our self-consistent calculations was 10^−4 ^eV.

## Results and Discussion

### Crystal structure and electronic structure of arsenene

**Figure 1:**
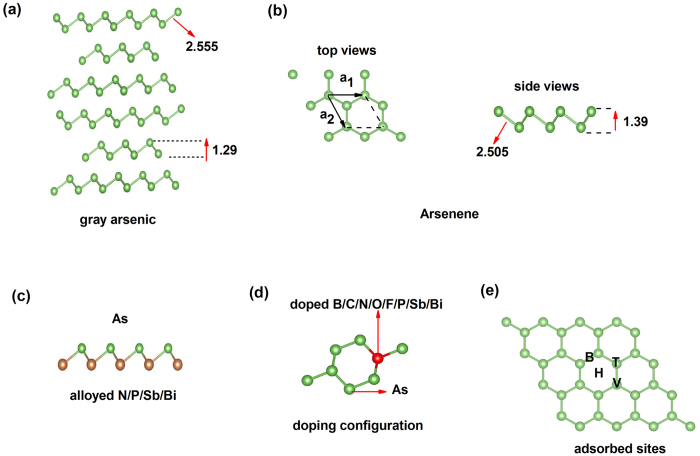
Geometric structures of (**a**) bulk gray arsenic, (**b**) top and side views of arsenene, (**c**) alloys of arsenene with As half substituted by N, P, Sb and Bi, (**d**) the doping configuration (arsenene doped with B, C, N, O, F, P, Sb and Bi atoms), (**e**) four different adsorption sites considered in arsenene.

We first calculated the structure characteristics of bulk gray arsenic and arsenene (monolayer gray arsenic), with the optimized structural models shown in Fig. 1a,b. For the bulk gray arsenic, the determined buckled heights, bond lengths and bond angles are 1.29 Ǻ, 2.555 Ǻ and 96.735°, respectively. However, in arsenene, due to the interlayer coupling disappeared, the corresponding buckled heights, bond lengths and bond angles are 1.39 Ǻ, 2.505 Ǻ and 92.097°, respectively. In comparison, the arsenene has higher buckled-heights, smaller bond lengths and larger bond angles for bulk gray arsenic. More importantly, from bulk gray arsenic changing to arsenene, the semimetal to wide-gap semiconductor transition has been revealed owing to second-order effects and quantum confinement effect^8^.

**Figure 2:**
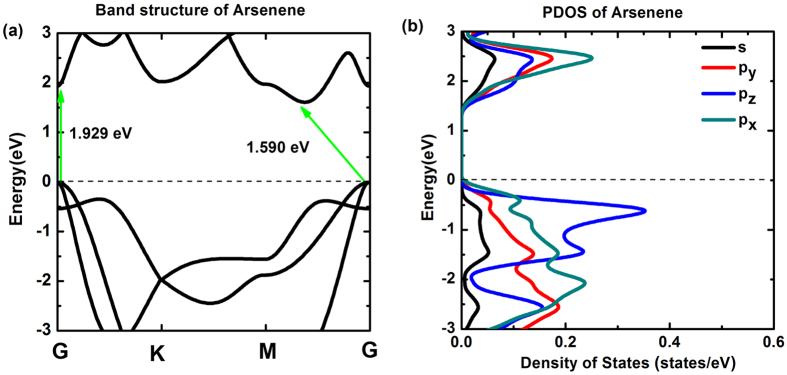
Electronic structure of arsenene by PBE method. (**a**) Band structure of arsenene, (**b**) the projected density of states (PDOS) of arsenene.

Next, we calculated the electronic structure of arsenene. The *E*(*k*) plotted in Fig. 2a, the valence band maximum (VBM) is located at the G point and the conduction band minimum (CBM) locates in the middle of the M-G direction. In the arsenene, the indirect band gap of 1.590 eV is narrow comparing to the 1.929 eV direct band gap at G point, so it is indirect band gap semiconductor. Under the HSE06 hybrid functional, the indirect band gap is increased to 2.323 eV. These results are nearly consistent with previous works^8^,^9^,^17^. It is noted that the states near the top of valence band at G point display strong dispersion, which means relatively low holes effective mass. As we will see later, the characters of CBM and VBM will be significantly altered by substituting As atom with N, P, Sb and Bi atoms. In the other hand, from the projected density of states (PDOS) of arsenene (Fig. 2b), the states near the Fermi level are mainly contributed by p orbitals, in particular to p_z_ and p_x_ orbitals, while the s orbitals make a relatively small contribution. The p orbitals have dominant contribution deriving from sp^2^ + sp^3^ hybridization, since sp^2^ hybridization is unstable when the bulk gray arsenic forms monolayer honeycomb structure^19^.

### Cohesive energy, structural parameters, phonon spectra and electronic structure of AsN, AsP, SbAs and BiAs alloys

**Figure 3:**
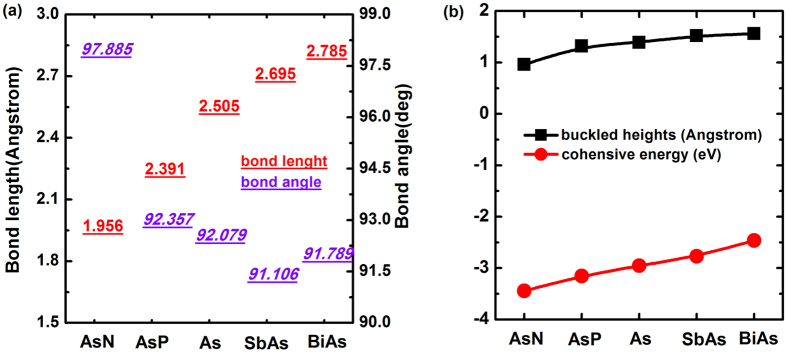
The changing trend of (**a**) bond lengths and bond angles, (**b**) buckled heights and cohesive energies from AsN to BiAs.

It is well known that the direct band gap is desirable with respect to the semiconductors applying to optoelectronic devices. For the sake of achieving direct band gap and averting structures destroyed, we substituted As atom with the same group elements N, P, Sb and Bi in the arsenene, as shown in Fig. 1c. Firstly, the cohesive energy of arsenene is calculated to examine its stability. The cohesive energy is defined by


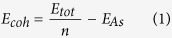


where *E*_*tot*_ is the total energy of arsenene; *n* is the number of As atoms and *E*_*As*_ is the energy of single As atom. The cohesive energy is −2.953 eV/atom, this result is nearly identical with previous work^17^. As a comparison, we define the cohesive energy of substituted systems, which is:^25^


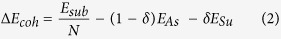


where *E*_*sub*_ is the total energy of arsenene half-substituted by N, P, Sb or Bi atoms; *δ* = *n*(*Su*)*/N*, *N* is the number of atoms in substituted systems and *n*(*Su*) is the number of substituting atoms; *E*_*As*_and *E*_*Su*_ are the total energy of single As atom and substituting atoms, respectively. The results of cohesive energy and optimized geometric parameters are listed in Table 1, Fig. 3 shows their differences more evidently.

**Figure 4:**
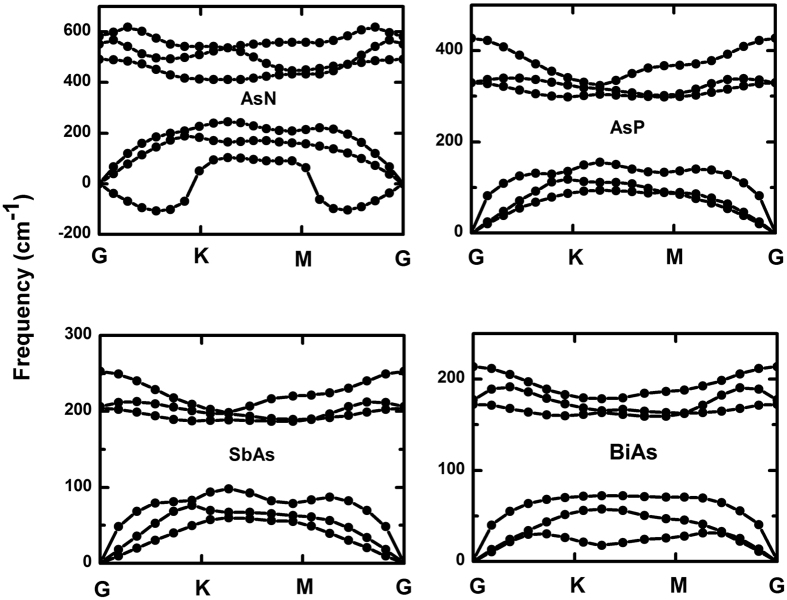
Phonon band dispersions of AsN, AsP, SbAs and BiAs alloys, which exhibit outstanding kinetic stability for AsP, SbAs and BiAs alloys.

The dynamic stability and experimental feasibility are very important properties of these systems. We thus studied the phonon spectra and performed an *ab initio* molecular dynamics (AIMD) simulation. As seen in Fig. 4, there are not soft phonon modes in AsP, SbAs and BiAs substituted systems, which demonstrates that these alloys are kinetically stable. In the case of AsN, the frequency of the transverse acoustic mode along G-K and G-M directions is negative due to the softening of phonons, indicating that AsN may be not kinetically stable. And AsP, SbAs and BiAs alloys may be feasible in experiment, for which the AIMDs suggest that these alloys always remain original structures from 300 K to 500 K with a time step of 3 fs and running the simulation for 300 fs. Furthermore, from Table 1, the cohesive energy for all substituted systems is negative, which confirms their thermal stability but AsN may be not kinetically stable. Particularly, an approximately linear increase of cohesive energy from BiAs to AsN is found with the electronegativity of substituting elements in Fig. 3b. Similarly, we also notice that the buckled heights increase monotonously with the electronegativity. However, the change of lattice constants is contrary to cohesive energy and buckled heights. This is due to the fact that, for the lattice constants, they are mainly affected by atomic radius instead of electronegativity. This case is also analogous to the tensile in-layer strain. As seen in Fig. 3a, from AsN to BiAs, the changing trend of bond lengths and bond angles is nearly opposite, but the bond angles have an abrupt raise in BiAs. Despite the fact that the electronegtivity will effectively induce the decrease of bond length, the effect of electronegtivity may be smaller than atomic radius for the bond angle since the bond angle of BiAs is larger than SbAs.

**Figure 5:**
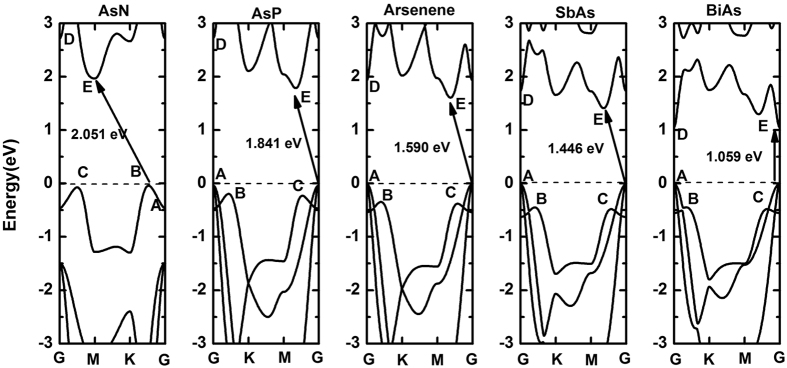
The band structure of AsN, AsP, Arsenene, SbAs and BiAs, an indirect band gap of AsN, AsP, Arsenene and SbAs are found and BiAs is direct band gap.

To further explore the properties of substituted systems, the calculated band structures are displayed in Fig. 5. The band gaps are widened when arsenene is substituted by N and P, which is opposite to the cases by Sb and Bi. Most notably, BiAs demonstrates direct band gap of 1.059 eV, namely we obtain an indirect-direct transition for arsenene by substituting half As atoms with Bi atoms. And the direct band gap of BiAs is increased to 1.728 eV under more precise HSE06 hybrid functional calculation. In order to indicate the process of indirect-direct transition more distinctly, we name the VBM at G as point “A”, two valence band maximum away from G as points “B” and “C”, CBM along M-G direction as point “E”, and local conduction band minimum at G as point “D”. By comparing all systems, the substitution of N or P results in the moving up of “D” and “E” points and increases the band gap. On the contrary, the “D” and “E” points are moved down with the substitution of Sb or Bi and the band gap decreases. In particular, for “A” point in AsN, its energy is smaller than “B” and “C” points, which is an important change of the frontier states caused by the separation of two nearest-neighbor valence bands near G. And for “D” point in BiAs, its energy is decreased and smaller than “E” point owing to the substitution of Bi, which produces the direct band gap semiconductor.

### Semiconductor-metal and indirect-direct band gap transitions by biaxial strain in alloyed systems

**Figure 6:**
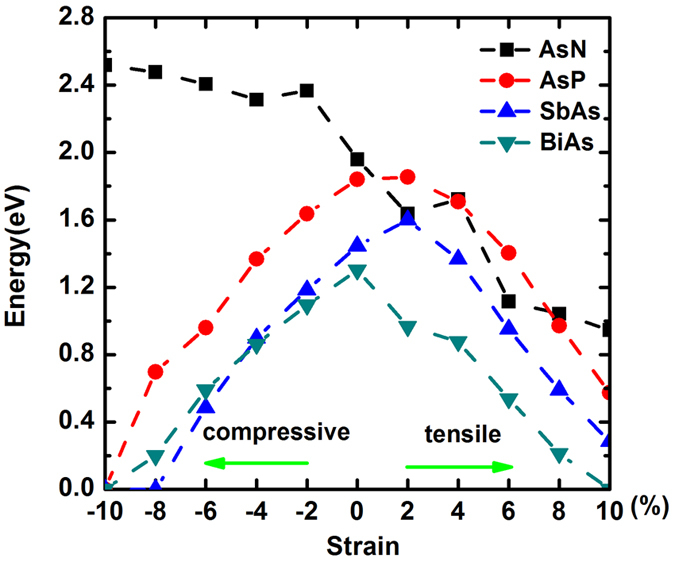
The dependence of the band gap *Eg* with the strain from −10% to 10% in four alloys.

The alloyed systems were also subjected to the in-plane biaxial strain, which was achieved by the relative change of the lattice constant. As seen in Fig. 6, we plot the dependence of the band gap *Eg* with the strain from −10% to 10% in four alloys. The −8% compressive strain in the SbAs gives rise to a semiconductor-metal transition, both AsP and BiAs have a same transition when the compressive strain increases to −10%. Moreover, the semiconducting BiAs will convert to metallic one by the 10% tensile strain. Especially, the AsN constantly retains the semiconducting character by changing strain from −10% to 10%, manifesting a superior strain property in AsN alloy. But, it should be mentioned that the application of AsN may be restricted owing to its kinetically unstable. In the alloyed systems, the strain not only gives rise to the semiconductor-metal transition, but also induces the indirect-direct band gap transition. Showing in Table 2, analogous to buckled arsenene, even though the fundamental band gap of AsP and SbAs is indirect, the indirect-direct transition appears under 8% and 6% tensile strain, respectively. We also find that the direct band gap of alloys can’t be induced by compressive strain, because “D” point is mostly affected by tensile strain. Hence the direct band gap locates at G. Moreover, the fundamental direct band gap can be converted to indirect band gap with ≥−2% compressive strain in BiAs alloy.

**Figure 7:**
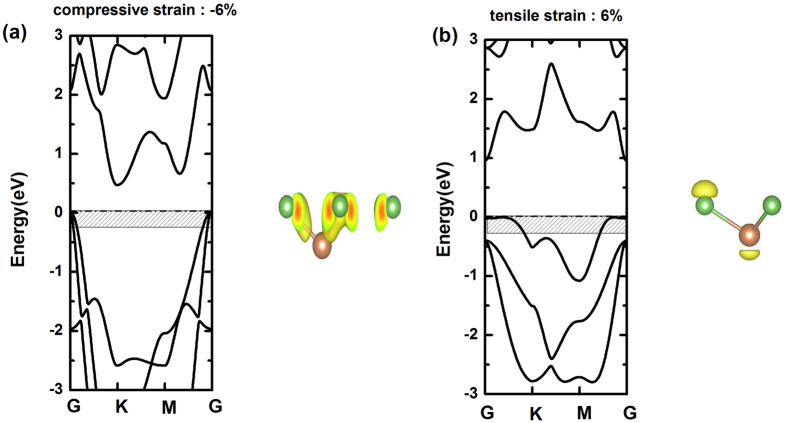
Electronic structure of SbAs subject to an in-plane biaxial strain of (**a**) −6% and (**b**) 6%. The energy range between the Fermi level and 0.25 eV below the top of the valence band is black shaded in the band structure in the left panels. The electronic density of these states superposing with a ball-and-stick model of structure is shown in the right panels.

Taking SbAs as an example, the character of the frontier states that locate near G is drastically affected by tensile strain which causes the indirect-direct band gap transition. As shown in Fig. 7a, the compressive strain of −6% reduces the band gap and makes the large dispersion of valence band near G, but keeps indirect band gap and the σ-like character of frontier states. As seen in Fig. 7b, we find the direct band gap of 0.95 eV with 6% tensile strain. When the tensile strain is ≥6%, the lower overlap of p orbitals between As atoms and Sb atoms results in the small dispersion of valence band near G corresponding the π-like character of frontier states, and the system exhibits a direct band gap character. The same situation is found for AsP with ≥8% tensile strain. Therefore, we expect that the results can make these alloys into outstanding candidates for the mechanical sensors in the future.

### Formation energies, structural parameters and electronic structures of the doped arsenene systems

For the doped systems, the doping configuration is illustrated in Fig. 1d. We calculated the formation energy of doped systems to confirm their thermal stabilities. The formation energy in doped systems is defined as:^26^


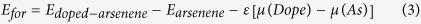


where *E*_*doped-arsenene*_ represents the total energy of doped arsenene and *E*_*arsenene*_ is the total energy of pristine arsenene; *ε* is the number of doped atoms transferred between a perfect cell and its reservoir forming a defect; *μ*(*Dope*) and *μ*(*As*) are the chemical potentials of doped atoms and As atoms, respectively. Optimized structure parameters and calculated formation energies, Fermi levels, band gaps for B, C, N, O, F, P, Sb and Bi doped in arsenene monolayers are summarized in Table 3, it obviously suggests that B-, C-, N-, O- and P-doped systems possess negative formation energy, indicating that they are relatively stable.

Nevertheless, the formation energy of F-doped buckled arsenene is positive, which is contrary to F-doped puckered arsenene^26^. The opposite sign of formation energy for F-doped buckled and puckered arsenene may derive from two differently typical honeycomb structures. Furthermore, the bond lengths and bond angles of these systems are smaller than that of pristine arsenene. Next, we focus on whether these doped systems can be realized in experiments. Hence, the stabilities of B-, C-, N- and O-doped systems were tested at finite temperature by performing an *ab initio* molecular dynamics (AIMD) simulation. The temperature of our simulation is from 300 K to 500 K with a time step of 3 fs and running the simulation for 300 fs. Our AIMD results reveal that these systems remain stable from 300 K to 500 K. The results reflect the stabilities of these systems, and they may be realized in experiments at room temperature.

**Figure 8:**
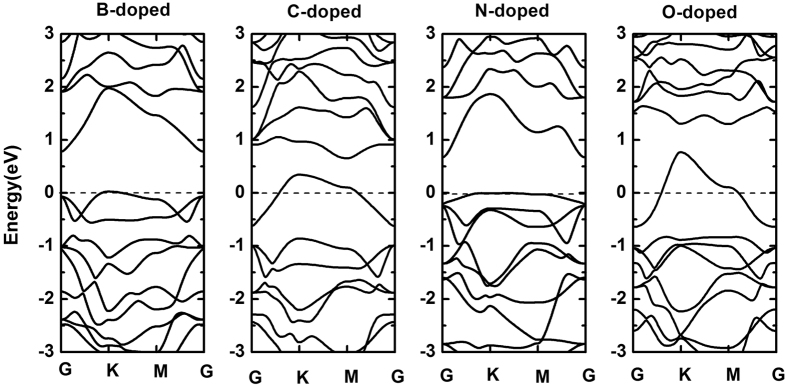
The band structure of B-, C-, N- and O-doped arsenene. B- and N-doped arsenene are semiconducting character and C- and O-doped arsenene are metallic character.

We plot the band structures of B-, C-, N- and O-doped systems in Fig. 8. It is easy to see that B- and N-doped systems show semiconducting character with indirect band gap of 0.76 eV and 0.68 eV, respectively. The band gap of N-doped system is apparently smaller than that of half As substituted by N in arsenene. Notably, the highest valence band is evidently unfilled which indicates metallic character for both C- and O-doped systems. At this point, the following question arose: why B- and N-doped systems exhibit semiconducting character, but C- and O-doped systems exhibit metallic character? We need to analyze the microscopic origin of their semiconducting and metallic character. In the pristine arsenene, each As atom forms covalent σ bonds with three nearest As atoms (bond angle 92.097°) and leaves one lone pair electrons, meaning that the bonds of pristine arsenene are saturated. In the case of B- and N-doped arsenene, each B and N atoms contributes three valence electrons to bond with three nearest As atoms. But B only has three valence electrons and the valence electrons of N are equal to As atom, which retains the saturated bonds of doped systems and exhibits the semiconducting character. However, for C- and O-doped systems, the saturated bonds of systems are missing. When C dopes in arsenene, three valence electrons of C bond with three nearest As atoms. One non-bonding electron is left and the Fermi level crosses the defect band. The system displays the metallic character. As similar as C-doped system, O-doped system also induces one non-bonding electron which attributes to only two valence electrons of O atom bonding with nearest two As atoms^26^. Thus, the highest valence band of C- and O-doped systems is unfilled and the defect band localizes at the Fermi level, since these systems form unsaturated metallic bonds.

**Figure 9:**
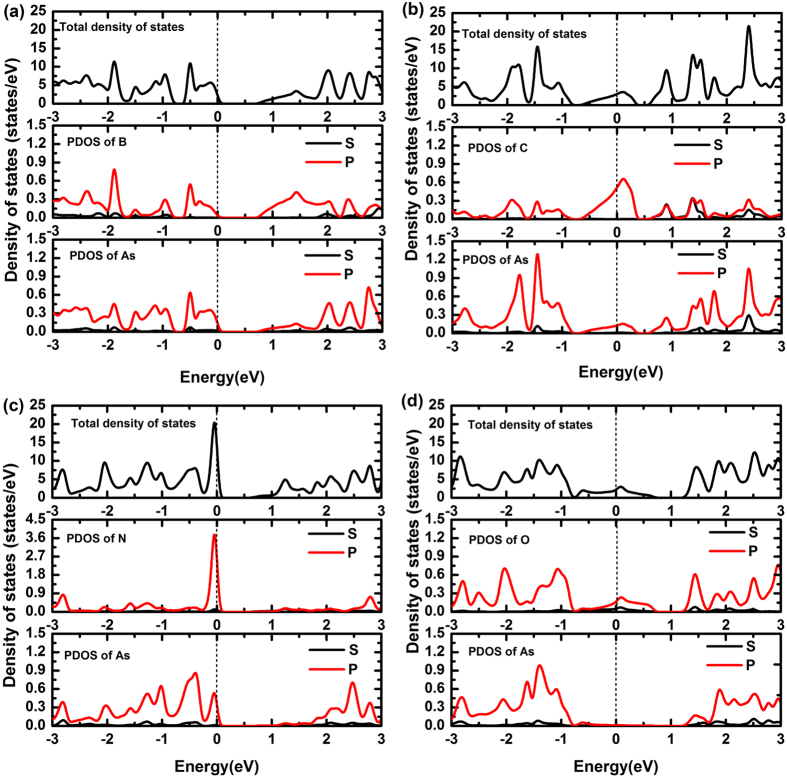
DOSs of arsenene (**a**) and (**c**) doped with B and N, exhibiting p-type semiconducting character, (**b**) and (**d**) doped with C and O, exhibiting metallic character.

The density of states (DOSs) of B-, C-, N- and O-doped arsenene are depicted in Fig. 9. It can be seen that B and N atoms act as p-type dopants because the Fermi level crosses to the frontier states of the highest valence band. In B-doped system, the frontier states are mainly contributed from the p orbitals of As atom. But in N-doped system, the frontier states are contributed from s and p orbitals of N atom and p orbitals of As atom. Therefore, B- and N-doped arsenene may be applied to p-type semiconductors. Unlike B and N dopants, C and O induce mid gap defect states. This is because C and As atoms contribute the non-bonding electrons in C-and O-doped systems, respectively. Notably, the states near the Fermi level in C-and O-doped systems mostly stem from the p orbitals of dopant atoms, which is different from B-doped system. The semiconducting-metallic transition induced by C and O dopants may be valuable for these systems applications in 2D electronic materials.

### The spin asymmetric and a bipolar spin-gapless-semiconducting character at the external E-field in the C-doped arsenene

**Figure 10:**
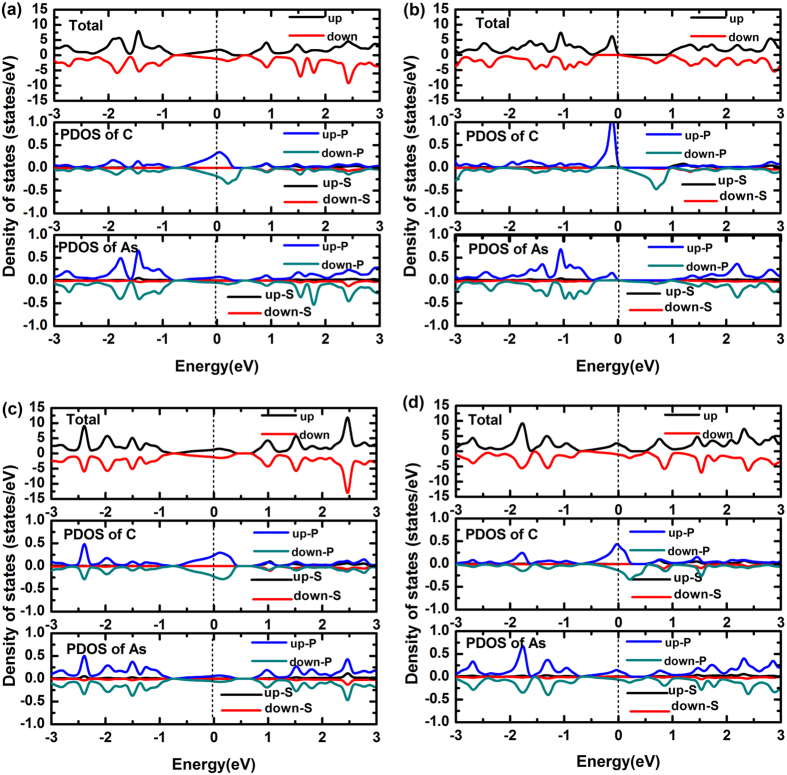
Spin-polarized DOSs of C-doped arsenene (**a**) intrinsic (**b**) at E-field of −0.32 V/nm, (**c**) with −2%, (**d**) with 2% strain.

According to the above results, C- and O-doped systems possess half-filled 2p bands. Thus we performed the spin-polarization calculations. As seen in Fig. 10a, C-doped system is spin asymmetric, and the spin-polarized states at the Fermi level mainly come from the p orbitals of C dopant. We also calculated the magnetic energy to explore the energy gain from the spin polarization. The magnetic energy is defined as


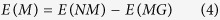


The *E*(*M*) is gained about 1 meV for C-doped system, this small value indicates that the magnetism may be easily influenced by the thermal fluctuation and external conditions^27^. Thus, we first consider the effect of strain. In Fig. 10c,d, we plot the DOSs of C-doped system under −2% compressive strain and 2% tensile strain. When −2% compressive strain is applied to C-doped system, the system is changed to be spin symmetric. But 2% tensile strain does not change the spin symmetry. This indicates that the compressive strain can sensitively mediate the spin property of C-doped system. In addition, the system always maintains the metallic character when the strain changes from −10% to 10%, displaying a superior strain property.

Generally speaking, the external electric field (E-field) can effectively tune the spin polarization. To probe into the electronic properties of C-doped arsenene, we applied an external E-field perpendicular to the C-doped arsenene plane. Owing to that the external electric field will increase the interaction energy of the half-filled 2p bands, the 2p states in C-doped atoms should enhance the spin-polarization to reduce the interaction energy. As shown in Fig. 10b, when the external E-field equals −0.32 V/nm, the spin-up channel is moved below the Fermi level, while the spin-down channel is moved above the Fermi level. We find that the system is gapless between spin-up and spin-down. Hence a bipolar spin-gapless-semiconducting character is obtained^27^. The states near the Fermi level primarily derive from the p orbitals of C atom, despite the fact that the p orbitals of As atom also have a little contribution. According to the above results, the C-doped arsenene may be applied to the spintronic devices in the future, since the E-field required for this transition is not quite high and it is possible to realize in experiment. However, for pristine buckled arsenene and C-doped puckered arsenene, tuning their electronic structures need a quite high external E-field (there are indirect-direct band gap transition at 4.2 V/nm for the pristine buckled arsenene and metal-half metal transition at 8 V/nm for the C-doped puckered arsenene)^19^,^26^.

**Figure 11:**
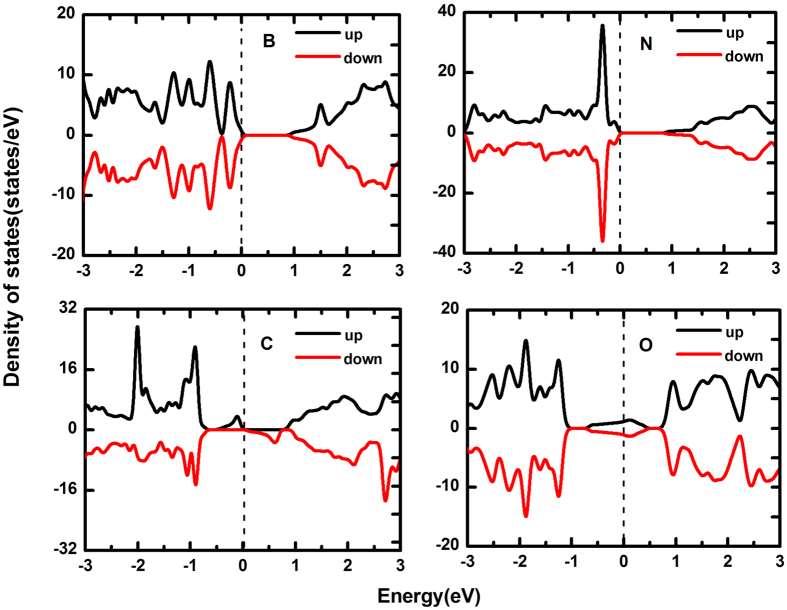
Spin-polarized DOSs of B-, C-, N- and O-doped arsenene with 5.56% doping concentration.

**Figure 12:**
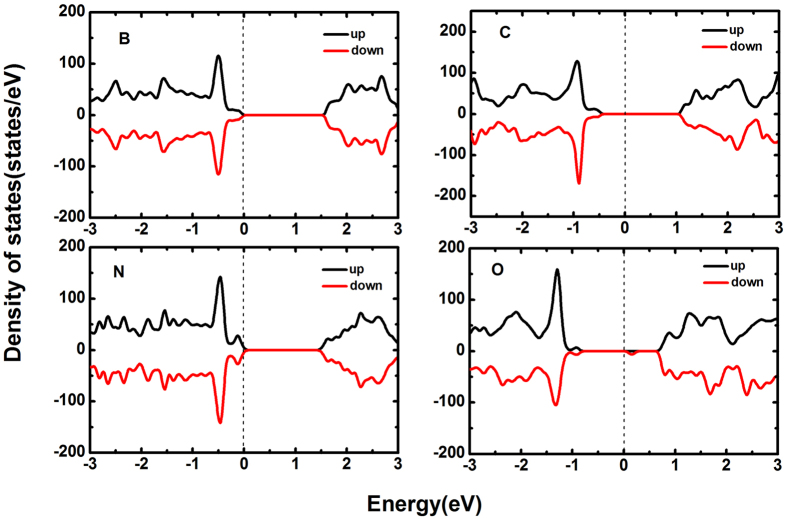
Spin-polarized DOSs of B-, C-, N- and O-doped arsenene with 0.78% doping concentration.

Moreover, we also studied the lower doping ratio cases (using 3 × 3 × 1, 4 × 4 × 1 and 8 × 8 × 1 supercells). Shown in Fig. 11, when the doping concentration is 5.56% (using 3 × 3 × 1 supercell), the results of B-, N- and O-doped systems are similar to the lager doping ratio cases. But, we notice that the C-doped system possesses a lager spin-polarization leading to an approximately bipolar spin-gapless-semiconducting character. Next, we further decrease the doping concentration (not plotted in here). As the doping concentration is decreased to 3.13% (using 4 × 4 × 1 supercell), B-, N- and O-doped systems are still analogous to the lager doping ratio cases and C-doped system possesses bipolar spin-semiconducting character with a 0.45 eV band gap. More importantly, a 1 μ_B_ magnetic moment is found in both 5.56% and 3.13% doping concentration, while the magnetic moment is only 0.25 μ_B_ in larger doping ratio cases. This result indicates that the doping concentration can effectively affect the magnetism of the C-doped system. Whether the relatively low doping concentration will change the above results, and then 0.78% doping concentration (using 8 × 8 × 1 supercell) is investigated, as shown in Fig. 12. No noticeable change is found in both B and N doping systems, conversely, C- and O-doped arsenene exhibit a novel character. The results in Fig. 12 clearly indicate C- and O-doped arsenene are semiconducting character with 1.46 eV and 0.76 eV band gaps, that is, using the relatively low doping concentration remains the intrinsic semiconducting character of arsenene. On the other hand, the C-doped arsenene remains a 1 μ_B_ magnetic moment even if the impurity states have removed when the relatively low doping concentration is used. Remarkably, there is a 1 μ_B_ magnetic moment produced in the O-doped arsenene and the spin-polarized states are contributed by O atom, which is not found in the above cases and predicts the relatively low doping concentration will be conducive to the spin-polarized asymmetry of O-doped arsenene.

**Figure 13:**
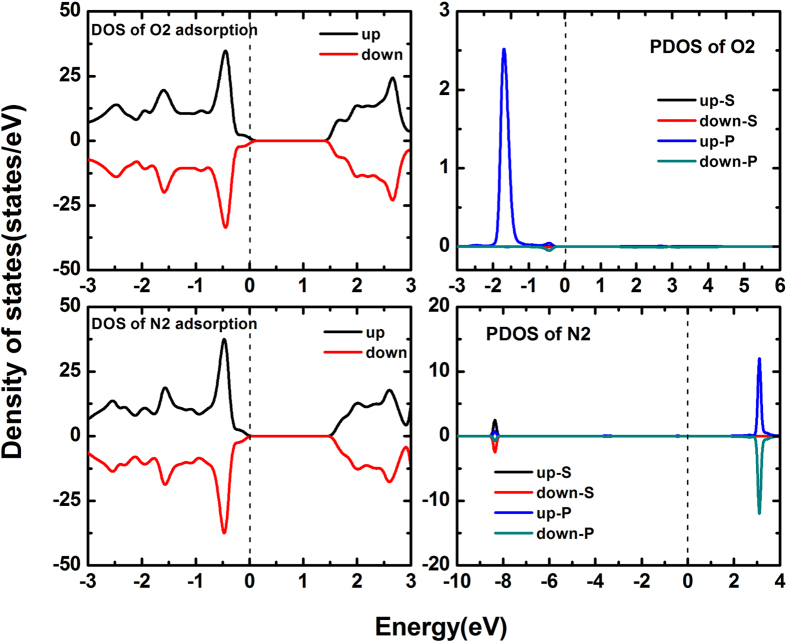
Spin-polarized DOSs of O2 and N2 adsorbed arsenene.

Finally, we briefly discuss the molecule adsorbed surface of arsenene. As shown in Fig. 1(e), there are four different adsorption sites considered in arsenene owing to the buckled hexagonal lattice structure, they are named as hill site (T), hollow site (H), valley site (V) and bridge site (B), respectively. We consider two gas molecules (O2 and N2) are adsorbed on the surface of arsenene, as consequence of O2 and N2 a relatively high content in the air. After the sufficient optimization, it has been obtained that O2 molecule prefers to adsorb hollow site and valley site is the most favorable adsorption site for the N2 molecule. The adsorption energy is also calculated to further study the adsorption property. The adsorption energy is defined as:


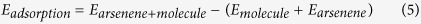


where 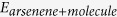 is the total energy of surface-adsorbed arsenene, 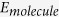 and 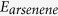 are the total energy of molecule and pristine arsenene, respectively. The calculated results show that the adsorption energy of O2- and N2-adsorbed arsenene are 0.150 eV and 0.573 eV, indicating this two molecules adsorbed on the surface of arsenene will be endothermic. Besides, the calculated adsorption heights are 3.10 Ǻ and 3.21 Ǻ for O2 and N2 adsorption. We attend that the above results are much larger than some light adatoms adsorbed on the arsenene nanosheets^28^, which means that the O2 and N2 molecules are not easy to be adsorbed on the surface of arsenene compared to the light atoms. Now we turn to focus on the electronic structure of molecule adsorbed arsenene. The DOSs of two adsorbed cases are plotted in Fig. 13. From the DOSs, both O2- and N2-adsorbed arsenene have about 1.50 eV band gap, but the O2 molecule induces a p-type adsorbing feature. In particular, the O2 molecule is spin asymmetric, which endows a 2 μ_B_ magnetic moment for the system. Indeed, the effect of O2 and N2 molecule adsorption is relatively weak for the electronic structure of arsenene because of the lager adsorption heights even though the magnetism can be induced by O2 molecule.

## Conclusion

In conclusion, we have predicted the equilibrium geometry and electronic structure of As-alloys and doped arsenene based on the DFT calculations. AsN, AsP and SbAs are semiconductors with indirect band gap and BiAs is direct band gap. It is also able to modulate the electronic structure of these systems over wide range by biaxial strain. Furthermore, an appropriate biaxial strain can induce the indirect-direct and semiconducting-metallic transitions in AsP, SbAs and BiAs. The tensile strain-induced indirect-direct transition can be attributed to the frontier states at the top of valence band changing from σ-like to π-like character. In addition, we find that B-, C-, N-, O- and P-doped systems are stable corresponding to negative formation energy. B- and N-doped systems possess p-type semiconducting character while C- and O-doped systems are metallic. Remarkably, the C-doped system is spin-polarization asymmetric, it will be changed to be spin-polarization symmetric under −2% compressive strain. The C-doped system will become a bipolar spin-gapless semiconductor at −0.32 V/nm external E-filed. And the doping concentration can effectively affect the magnetism of the C-doped system and the relatively low doping concentration will be conducive to the spin-polarized asymmetry of O-doped arsenene. For the molecule adsorbed arsenene, the adsorption process is endothermic and the adsorption height is obviously lager than light atoms adsorption, which mean the effect of O2 and N2 molecule adsorption is relatively weak for the electronic structure of arsenene. Our results will be evaluable to the arsenene sheets applying to the mechanical sensors and spintronic devices in the future.

## Additional Information

**How to cite this article**: Liu, M.-Y. *et al.* Unexpected electronic structure of the alloyed and doped arsenene sheets: First-Principles calculations. *Sci. Rep.* **6**, 29114; doi: 10.1038/srep29114 (2016).

## Figures and Tables

**Table 1:** The results for optimized geometries and cohesive energy of arsenene and its alloys obtained using DFT with the PBE exchange-correlation functional.

	Lattice constants (Ǻ	Cohesive energy (eV/atom)	Bond length (Ǻ)	Bond angle (deg)	Buckled heights (Ǻ)
AsN	3.0	−3.443	1.956	97.885	0.96
AsP	3.5	−3.153	2.391	92.357	1.32
Arsenene	3.6	−2.953	2.505	92.097	1.39
SbAs	3.8	−2.771	2.695	91.160	1.52
BiAs	4.0	−2.642	2.785	91.789	1.56

**Table 2:** The band gap *Eg*(eV) of four alloys changes with strain from −10% to 10%, the superscripts *d* and *i* represent the direct band gap and indirect band gap, respectively.

	−10%	−8%	−6%	−4%	−2%	0	2%	4%	6%	8%	10%
AsN	2.52^***i***^	2.48^***i***^	2.41^***i***^	2.31^***i***^	2.37^***i***^	1.96^***i***^	1.64^***i***^	1.72^***i***^	1.11^***i***^	1.04^***i***^	0.95^***i***^
AsP	0	0.70^***i***^	0.96^***i***^	1.37^***i***^	1.64^***i***^	1.84^***i***^	1.85^***i***^	1.71^***i***^	1.40^***i***^	0.97^***d***^	0.57^***d***^
SbAs	0	0	0.49^***i***^	0.90^***i***^	1.18^***i***^	1.44^***i***^	1.60^***i***^	1.37^***i***^	0.95^***d***^	0.59^***d***^	0.28^***d***^
BiAs	0	0.20^***i***^	0.59^***i***^	0.86^***i***^	1.10^***i***^	1.30^***d***^	0.97^***d***^	0.88^***d***^	0.54^***d***^	0.21^***d***^	0

**Table 3:** Optimized structural parameters and calculated formation energies, Fermi levels, band gaps for B, C, N, O, F, P, Sb and Bi doped in arsenene monolayers.

	Formation energy (eV)	Fermi level (eV)	Band gap (eV)	Bond length (Ǻ)	Bond angle (deg)
B	**−**1.012	**−**3.51	0.76	2.070	114.881
C	**−**1.212	**−**2.78	0	1.991	114.595
N	**−**0.494	**−**3.66	0.68	2.041	102.818
O	**−**0.034	**−**3.14	0	2.143	113.584
F	0.576	**−**3.75	1.77	2.470	118.622
P	**−**0.262	**−**3.58	1.69	2.410	93.777
Sb	0.853	**−**2.84	1.24	2.659	86.803
Bi	1.227	**−**2.56	1.10	2.738	82.854
Arsenene	/	**−**3.65	1.59	2.505	92.097
